# New Approaches in Flexible Organic Field-Effect Transistors (FETs) Using InClPc

**DOI:** 10.3390/ma12101712

**Published:** 2019-05-27

**Authors:** María Elena Sánchez-Vergara, Leon Hamui, Sergio González Habib

**Affiliations:** Facultad de Ingeniería, Universidad Anáhuac México, Ave. Universidad Anáhuac 46, Col. Lomas Anáhuac, Huixquilucan P.C. 52786, Mexico; elena.sanchez@anahuac.mx (M.E.S.-V.); sergiohabib15@gmail.com (S.G.H.)

**Keywords:** flexible film, electronic device, electrical properties

## Abstract

Organic semiconductor materials have been the center of attention because they are scalable, low-cost for device fabrication, and they have good optical properties and mechanical flexibility, which encourages their research. Organic field-effect transistors (OFETs) have potential applications, specifically in flexible and low-cost electronics such as portable and wearable technologies. In this work we report the fabrication of an InClPc base flexible bottom-gate/top-contact OFET sandwich, configured by the high-evaporation vacuum technique. The gate substrate consisted of a bilayer poly(ethylene terephthalate) (PET) and indium–tin oxide (ITO) with nylon 11/Al_2_O_3_. The device was characterized by different techniques to determine chemical stability, absorbance, transmittance, bandgap, optical properties, and electrical characteristics in order to determine its structure and operational properties. IR spectroscopy verified that the thin films that integrated the device did not suffer degradation during the deposition process, and there were no impurities that affected the charge mobility in the OFET. Also, the InClPc semiconductor IR fingerprint was present on the deposited device. Surface analysis showed evidence of a nonhomogeneous film and also a cluster deposition process of the InClPc. Using the Tauc model, the device calculated indirect bandgap transitions of approximately 1.67 eV. The device’s field effect mobility had a value of 36.2 cm^2^ V^−1^ s^−1^, which was superior to mobility values obtained for commonly manufactured OFETs and increased its potential to be used in flexible organic electronics. Also, a subthreshold swing of 80.64 mV/dec was achieved and was adequate for this kind of organic-based semiconductor device. Therefore, semiconductor functionality is maintained at different gate voltages and is transferred accurately to the film, which makes these flexible OFETs a good candidate for electronic applications.

## 1. Introduction

**Figure 1:**
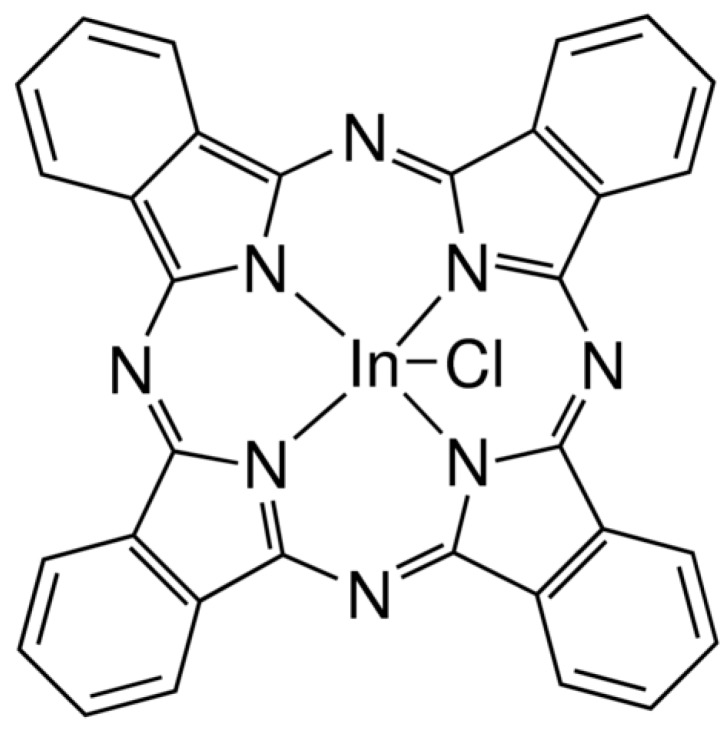
Structure of the InClPc.

In recent years, there has been a tendency towards the research of sustainable, organic field-effect transistors (OFETs), as inorganic materials have been exploited. OFETs have potential applications, specifically in research on flexible and low-cost electronics [1] such as portable and wearable technologies. The width and length of the channel located between the source electrodes and the collector, as well as the relative arrangement of the electrodes with respect to the semiconductor, will condition the operation of the device. Nanolamination is central in the fabrication of thin and uniform films for OFETs, in which high evaporation vacuum techniques are distinguished. These techniques provide a thin film with high uniformity, electrical performance, and electrical stability [2] that reduces the fabrication costs of these types of organic semiconductor devices. OFETs are the main units of electronic circuits, and their main function is to act as a switch or current amplifier that can be controlled by varying the gate voltage. For the last five decades, organic semiconductor materials (organic molecules and polymers) have been at the center of attention and investigation because of their optical properties, mechanical flexibility, and potential to fabricate large-scale and low-cost devices [3]. Phthalocyanines (Pcs) have certain characteristics that distinguish them from other organic semiconductors. This organic material has low optical losses, a nonlinear behavior, and low dielectric constants with picosecond response times [4]. Organic nonlinear optical materials such as Pcs have several applications such as in data storage, light modulation, and holographic devices [5]. Also, its manufacture and processes can be integrated and scalable on optical and electronic devices in a more economical and easier way than inorganic materials, which is desirable for the industry [6]. Recently, there has been an increase in demand of optical limiting (OL) material production because these materials are able to attenuate light intensity. Compared to nickel and zinc phthalocyanines, indium(III) phthalocyanine has a high optical limiting performance, as it has a relatively low limiting threshold, and a large nonlinear absorption coefficient [7]. Furthermore, Ruzgar et al. observed a 20-fold increase in field-effect mobility by fabricating a poly(vinyl alcohol) (PVA)/SiO_2_ bilayer gate dielectric for a copper(II) phthalocyanine (CuPc)-based OFET [8]. The latter indicates that the use of bilayer gate dielectrics plays a key role in the improvement of the device’s performance [9]. On the other hand, SiO_2_ is the most widely used dielectric. However, there is a constant search for alternative dielectrics, by the combination of different materials, in order to manufacture high-performance electronic devices on flexible substrates with cheap manufacturing techniques and reduced OFET operating voltages (the latter in order to increase or improve their properties and generate several applications in electronics [10,11]). There is a tendency for fabricated organic electronics devices to be highly flexible and comfortable, in which a clear example is the fabrication of “epidermal electronics” or “tattoo electronics” in the biomedical area to place on patients [12,13,14,15]. Organic electronic devices are characterized by their well-known flexibility and ease of transfer to different surfaces. The high conformability of these devices allows its adhesion on complex surfaces without a significant change or loss of electrical performance, which makes them important for advanced monitoring system applications in medical and sport areas [16]. It has amazing characteristics such as excellent impact and tensile strength, flexibility, transparency, and thermal stability [17]. Poly(ethylene terephthalate) (PET) properties such as strain, ductility, and flexibility tend to increase whenever the thickness of the film is decreased [18]. In addition to these characteristics, PET is a very easy material to recycle and has a low cost [19] with the current technologies. Thus, the base of many flexible OFET devices is poly(ethylene terephthalate) substrates with an indium–tin oxide (ITO) thin film [20]. Recently, optoelectronic devices have used thin ITO because of its characteristics such as high transparency and conductivity. Because they are flexible and lightweight, some applications of these materials are in flexible optoelectronic light-emitting diodes (LEDs) and touch screen devices [21]. Machinga et al. observed that ITO/PET substrates showed increasing compressive stress as the annealing temperature increased [20]. Therefore, these devices can be used in intermediate operation temperature applications. The present article aimed at the fabrication of a low-cost, flexible OFET device with a bilayer structure by using the high-evaporation vacuum technique in a bottom-gate/top-contact sandwich configuration, where the gate and substrate consisted of a bilayer PET with ITO (which makes the device flexible and thin). In addition, a nylon 11 and Al_2_O_3_ layer worked as the dielectric material, indium(III) phthalocyanine chloride (see Figure 1) acted as the semiconductor, and Ag ink dots acted as the source and drain of the OFET device. It is important to mention that Ag dots also were used as gate terminals for the device. The device was subjected to different characterization techniques in order to determine its structure and properties. Certain properties were determined including OFET chemical stability, absorbance and transmittance, optical properties, bandgap calculation, and the device’s electrical characteristics. A successful deposition of nylon 11 and InClPc with a bandgap of 1.67 eV and a maximum charge mobility of 36.2 cm^2^ V^−1^ s^−1^ was achieved, which made it a good candidate for flexible electronic applications.

## 2. Materials and Methods

**Figure 2:**
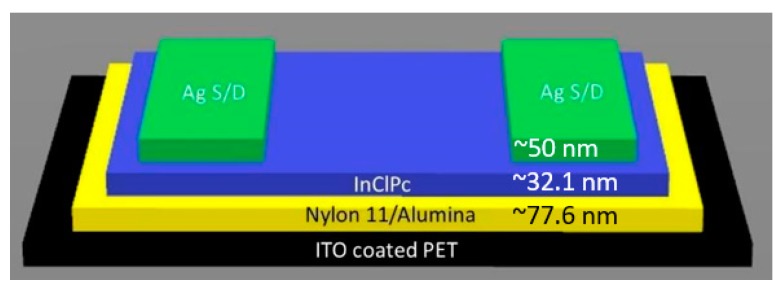
Organic field-effect transistor (OFET) after deposition in a bottom-gate/top-contact structure: (i) Ag source and drain dots at the top, (ii) InClPc, (iii) Nylon 11-Al_2_O_3_, and (iv) indium–tin oxide (ITO)-coated poly(ethylene terephthalate) (PET).

Commercial precursors were indium(III) phthalocyanine chloride (InClPc), Al_2_O_3_, and Nylon 11 (Sigma-Aldrich, Saint Louis, MO, USA), and they were used unpurified. In Figure 2, we can observe the bottom-gate/top-contact structure of the manufactured OFET, which consisted of (i) an organic semiconductor InClPc film, (ii) a dielectric film composed of Al_2_O_3_ particles embedded in a nylon 11 polymeric matrix, and (iii) gate, collector, and source electrodes. The electrode sources and collector were made of Ag ink dots. The electrodes’ functions were to inject charges in the organic semiconductor. According to Figure 2, the InClPc was isolated electrically from the electrode gate by the dielectric and on direct contact with the electrode source and collector. The bottom-gate/top-contact configuration was chosen because it usually presented lower contact resistances since the major contact surface existed between the electrodes and the organic semiconductor. Furthermore, manufacturing of the OFET started with the preparation of the PET substrate with an indium–tin oxide (PET-ITO) gate electrode. At the second stage of the process, thin films of the dielectric and the semiconductor were deposited. The deposition process was done in two stages using high-evaporation vacuum equipment with molybdenum boats. An evaporation rate of 1.4 Å/s, a temperature of 298 K, and a pressure 1 × 10^−6^ Torr were selected and maintained for the deposition process. Deposition film thicknesses were monitored using a quartz crystal monitor, which achieved ~77.6 nm for the nylon 11/alumina and ~32.1 nm for the InClPc. It is worth to mention that the dielectric film was constituted by Al_2_O_3_ particles within a nylon 11 polymeric matrix, which were submitted to thermal annealing after deposit. As a consequence of the latter, the polymeric fibers allowed particles to access the matrix. After deposition was done, thermal annealing took place at 150 °C for 30 min. After the thermal annealing process, a 12 °C/min cooling rate to room temperature for the samples was selected in order to close the nylon 11 fibers and to trap the Al_2_O_3_ particles inside the matrix. Afterwards, Ag dot electrodes 50 nm in thickness (source and drain) were deposited by hand using a thin brush and were left to dry for 24 h before characterization.

As shown in Figure 2, the OFET final conformation was PET/ITO/Nylon 11-Al_2_O_3_/InClPc/Ag with a sandwich type bottom-gate/top-contact structure configuration, which was characterized as follows. The chemical composition and purity of thin films that integrated the OFET was characterized by IR spectroscopy. Additionally, the crystalline structure in the MPc was verified by IR spectroscopy. The Fourier-transform infrared spectroscopy (FTIR) was conducted with a Nicolet spectrometer (Thermo Fisher Scientific Inc., Waltham, MA, USA). A Unicam spectrophotometer (Thermo Fisher Scientific Inc.) model UV300 was used to measure the optical absorption in the wavelength range of 200–1000 nm. Morphological characterization was done using a ZEISS EVO LS 10 scanning electron microscope (SEM) (Cambridge, UK). This equipment was operated using quartz substrate with a 10 kV voltage and a 25 mm focal distance. For electrical characterization of the OFET, a programmable voltage source, a Next Robotix sensing station (Comercializadora K Mox, S.A. de C.V., Benito Juárez, México City, Mexico), and a 4200-SCS-PK1 pico-ammeter auto-ranging Keithley (Tektronix Inc., Beaverton, OR, USA) were employed. For surface and morphological characterization, an optical Motic BA210 microscope with a Moticam 2.0 MP camera (New York Microscope Company Inc., Hicksville, NY, USA) was used. These characterizations were performed under normal conditions with a 40× magnitude. The process involved four different area analyses of the OFET to verify homogeneity of the films. Every area was monitored at normal conditions with normal room light and under ultraviolet (UV) light of 360 nm wavelength. Micrographs were taken for both conditions of the four areas mentioned previously. For the micrographs done under normal conditions and room light, a bar scale of 500 μm was used to make further analysis of the OFET surface. Additionally, for the micrographs done under dark conditions and UV light, an analysis of how much luminescence was presented in the OFET was done. The percentage of luminescence of each of the four areas was obtained in order to verify the quality along the films.

## 3. Results and Discussion

**Figure 3:**
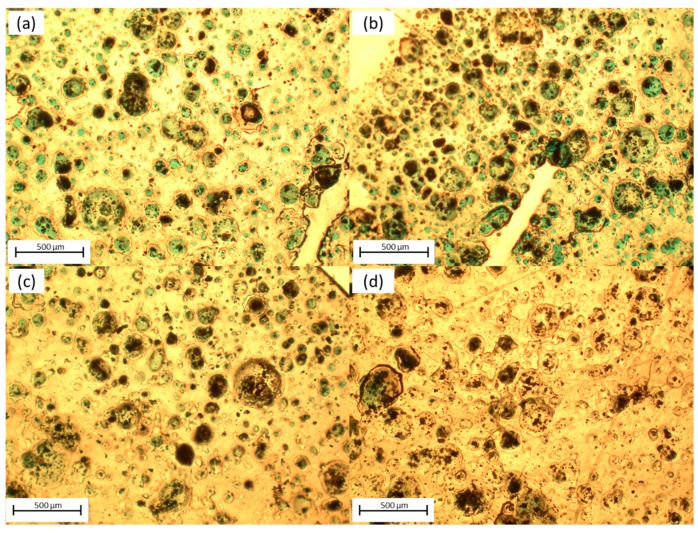
OFET optical characterization under normal light conditions. (**a**) Region 1; (**b**) Region 2; (**c**) Region 3; (**d**) Region 4.

In order to conduct surface morphology analyses, optical microscopy was used. Figure 3 shows four different region micrographs at 40× magnitude to unveil the surface changes along the sample. The presented regions were selected randomly to visualize film homogeneity. First, the surface showed dark and light regions. Rounded clusters were observed, mostly dark colored, embedded in the lighter region with different cluster sizes (24.3–361.5 μm). For the first two regions, most clusters presented a green color as the inner ring and some fully colored. For the next region, a lighter green color was observed in a few clusters, and for the last region only a few large clusters were light green colored. Surface roughness varied along the film, and clusters were subject to have darker areas than that of the lighter surface because of the increased thickness and the possible increase in the concentration of the deposited InClPc. Also, the green color was attributed to the InClPc, which indicated that the deposition process generated InClPc clusters, and the clusters increased further in size with the time of deposition and following thermal annealing. Nevertheless, different film homogeneity and equal cluster density were observed among the different regions, but each region had good film uniformity. Also, the clusters sizes varied for the different regions, as observed in Figure 3. In Region 1 the majority of the clusters had an intermediate size (~132 μm) and a few large clusters (~322 μm), and for Region 2 the clusters had mainly an intermediate size (~102 μm) and a few large clusters (~266 μm). For Regions 3 and 4, small clusters were abundant with sizes between 25–34 μm and a few intermediate clusters with sizes between 92–167 μm. The latter were related to the film deposition process and evidenced the amorphous structure of the device films. Furthermore, some film surface damage and roughness variations were observed.

**Figure 4:**
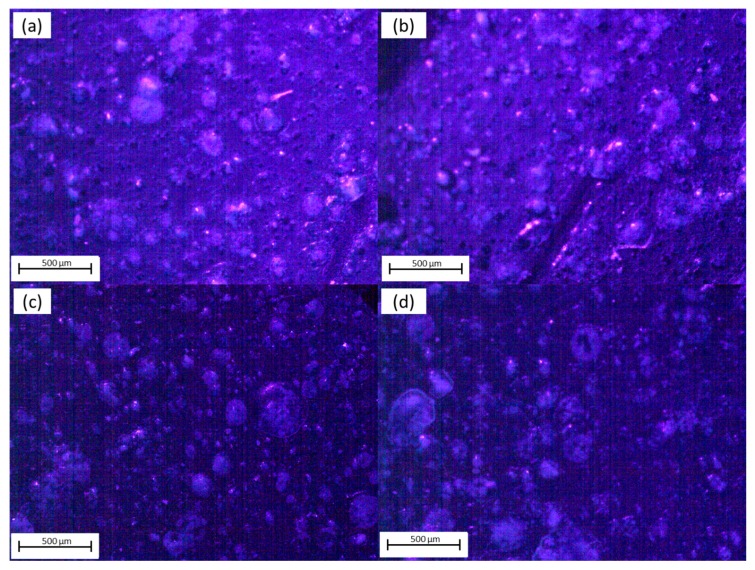
OFET optical characterization under UV light conditions. (**a**) Region 1; (**b**) Region 2; (**c**) Region 3; (**d**) Region 4.

Figure 4 shows the same four regions mentioned in Figure 3 under UV illumination. The latter was conducted to show the fluorescence and quality of the films. Fluorescence observed on the OFET surface was related to Al_2_O_3_ emission under UV light. Furthermore, rounded shaped clusters were still observed. Exposure to the UV light showed the film had regions that were thinner throughout its surface and contained porosity and some defects. For Regions 1 and 2 there were fluorescence emissions of the surface and clusters, whereas for Regions 3 and 4 the emissions were mainly related to the clusters. Darker areas showed that the film had clusters with higher emissions, which indicated that the InClPc also had a small contribution to the fluorescence observed. When exposed to UV light, the surfaces in the different regions had a high roughness, and different heights and depths were appreciated. Nevertheless, the mentioned clusters were observed to be homogeneously distributed on the film.

Table 1 show the fluorescent density % for the different OFET region micrographs presented in Figure 4. The latter allowed us to indirectly quantify the deposited InClPc concentration on the films. To obtain these results, surface luminosity intensity was quantified on the micrograph area (Figure 4), with respect to the total, as the surface was illuminated with a UV lamp. A higher value was observed in Region 2, approximately 5.6%, and a lower value of 0.6% was observed in Region 3 with an average fluorescent density of 2.48%. Moreover, Regions 3 and 4 presented lower values of fluorescent density, which, as mentioned previously, were related to InClPc cluster emissions and indirectly evidences the cluster density within the areas shown in Figure 3 and Figure 4.

**Figure 5:**
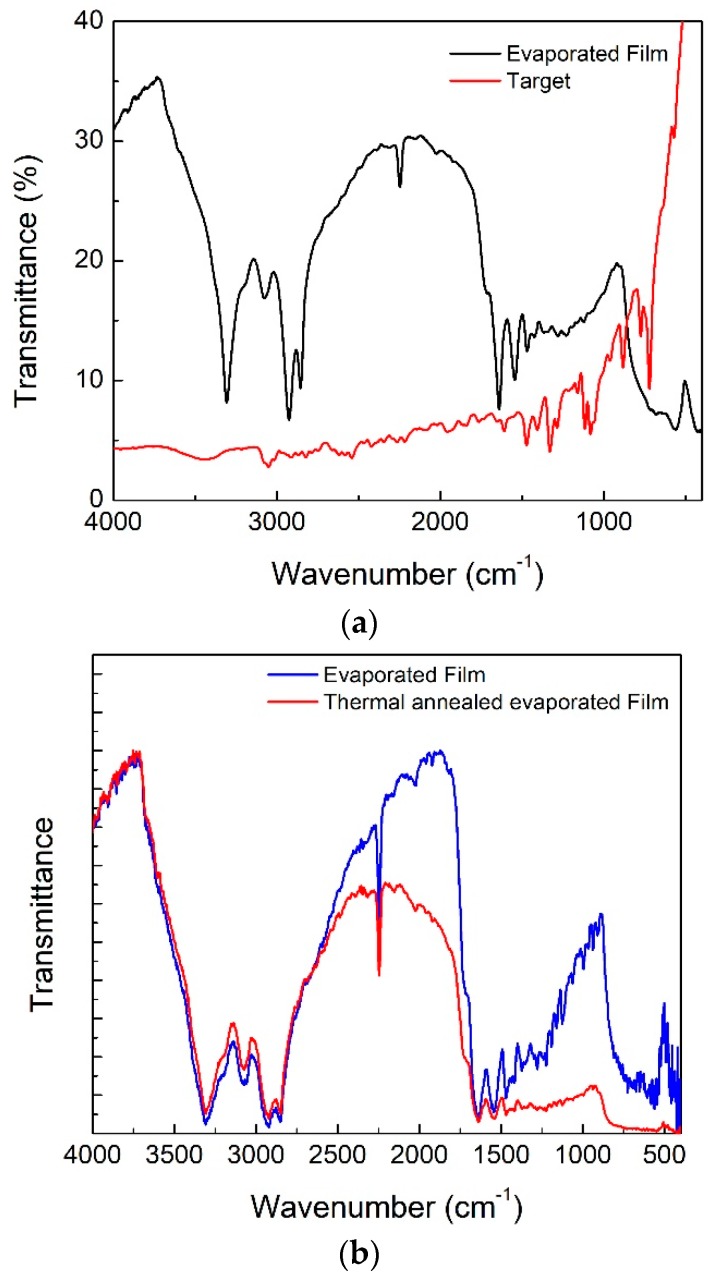
FTIR spectra of (**a**) the fabricated target and evaporated film and (**b**) the evaporated and thermal annealed films.

In Figure 5 the FTIR spectra before and after the high-vacuum evaporation technique for an InPc-KBr pill (target) and a film deposited on the monocrystalline silicon substrate were observed. As described before, a target was made in order to transfer the materials to a thin film with an evaporation process. The FTIR spectrum shown in Figure 5 provided evidence of the structural branched MPc and the success of the evaporation process. Peaks were observed for C=C_arom_ (~1605 cm^−1^), the cyano end group (2230 cm^−1^), and the aromatic CH (3070 cm^−1^). The 3410 cm^−1^ peak was mainly related to the OH moisture. These results indicated that the InClPc was successfully deposited. Additionally, the alpha (α) and beta (β) crystalline forms of the InClPc were observed at 777 and 720 cm^−1^, respectively [22,23]. Based on these results the MPc semiconductor did not suffer degradation during its evaporation and deposition processes because its characteristic IR signals appeared in the film’s spectrum. On the other hand, a change from a crystalline to an amorphous structure was observed. Initially, the MPc presented both crystalline structures α and β, but after the deposition, it suffered a change to an amorphous structure. This behavior was expected as a consequence of the quick phase changes that occurred in the InClPc during evaporation and deposition, which prevented nucleation and growth of ordered structures within the film. Furthermore, the stretching vibrations for NH had bands around 3308 and 3077 cm^−1^, and C=O vibrations in the polymeric matrix were around 1652 cm^−1^. These results indicated that nylon 11 was successfully deposited by high-vacuum evaporation as observed in previous works [24,25,26,27]. Furthermore, a thermal annealing was conducted for film relaxation, and the FTIR comparison spectra were observed in Figure 5b for the evaporated film and after annealing. The previously mentioned bands for the InClPc and nylon 11 were shown after thermal annealing. Also, a slight change in the FTIR spectra was observed. The latter indicated that thermal annealing allowed relaxation of the film structure without degrading nylon 11 or the InClPc. Therefore, the manufacturing process of the nylon 11-Al_2_O_3_/InClPc flexible OFET by high-vacuum evaporation followed by thermal annealing was adequate.

**Figure 6:**
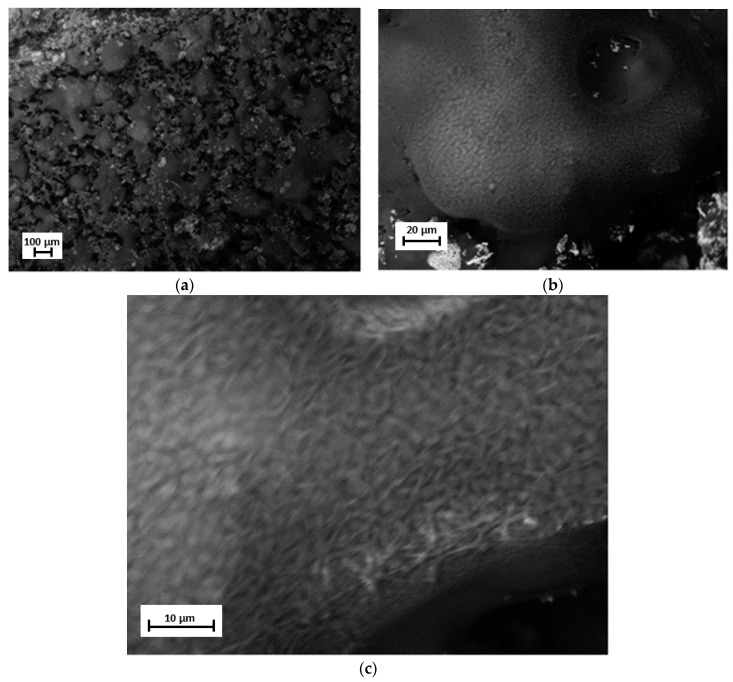
SEM micrographs of the film’s surface showing the nylon 11 fibers. (**a**) 150×; (**b**) 1.55 K×; (**c**) 4.04 K×.

SEM measurements were conducted in order to analyze the film’s surface morphology and to validate film deposition by the high-vacuum evaporation technique. Figure 6 shows the film surface morphology, where high roughness and an island-type deposit can be observed. Also, white acicular clusters with different sizes and orientations were randomly distributed on the film’s surface. In addition, circular pores with smooth topographies were observed and directly related to what was shown in Figure 3. Higher augments showed small fibers that were homogeneously distributed, with various orientations and lengths, on the film’s surface. These fibers were related to nylon 11, which indicated that the transfer of nylon 11 to the film by the evaporation process was successful.

**Figure 7:**
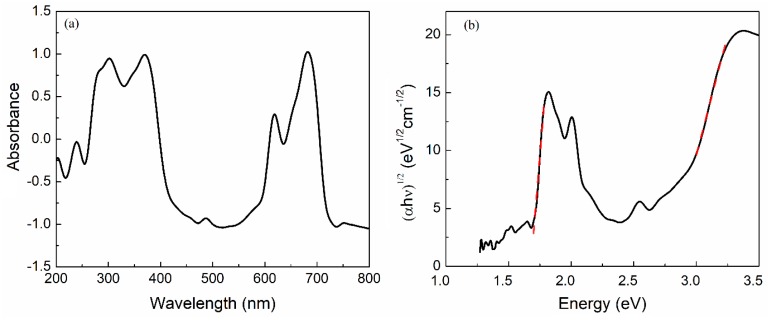
(**a**) UV-Vis of the flexible OFET and (**b**) Tauc Plot.

The UV-Vis absorbance spectra of the flexible OFET is shown in Figure 7a, where two principal bands can be observed. Electronic transitions between the HOMO and LUMO molecular orbitals were responsible for the intense Q and Soret bands in the visible and UV regions, respectively. These bands were related to the InClPc of the OFET and agreed with IR results [28]. Two peaks for the Q band (~620 and 680 nm) were observed in the visible range [22,29,30]. Which were related to the first π-π* transition on the InClPc semiconductor and to the second π-π* transition, vibrational internal intervals, surface states and excitation peaks, respectively [22,30]. The Soret band was within the UV region of the spectrum, around 340 nm [22,29,30], and was associated with molecules in electronic n-π* transitions. Additionally, a band gap was obtained from the UV-Vis absorption spectrum, using the Tauc model [31], by the intercept of (αhν)^1/2^ in the linear trend extrapolation, which was observed in the spectra by the abscissa axis. The absorption coefficient (α) usually obeyed the empirical relation α hν = β (hν − Eg)^n^ for amorphous semiconductors, where optical transitions were described by indirect transitions (n = 2) and β was the band edge parameter [31]. Using the Tauc model to obtain the optical bandgap, we were able to validate the device’s optical properties and relate them to OFET characteristics. The latter indicated that the device might perform different types of applications depending on its optoelectronic properties. A low bandgap implied greater intrinsic photogenerated carriers, and a high bandgap implied a selectively higher photon energy absorption. As shown in Figure 7b, two band gaps were obtained using the Tauc model represented by the red dashed slopes. Applying this model, the device’s indirect transitions were obtained, resulting in 1.67 eV, whilst for direct transitions the obtained value was 2.77 eV. These results indicated that there were different absorption processes for the fabricated semiconductor device. Nevertheless, because the amorphous structure of the semiconductor film integrated the OFET, it was expected that the dominant optoelectronic transitions were indirect, thus, a lower band gap energy was obtained. It should be mentioned that the band gap values for direct transitions were also within the ranges established for organic semiconductors, with band gap values between 1.5 and 4.0 eV [32].

**Figure 8:**
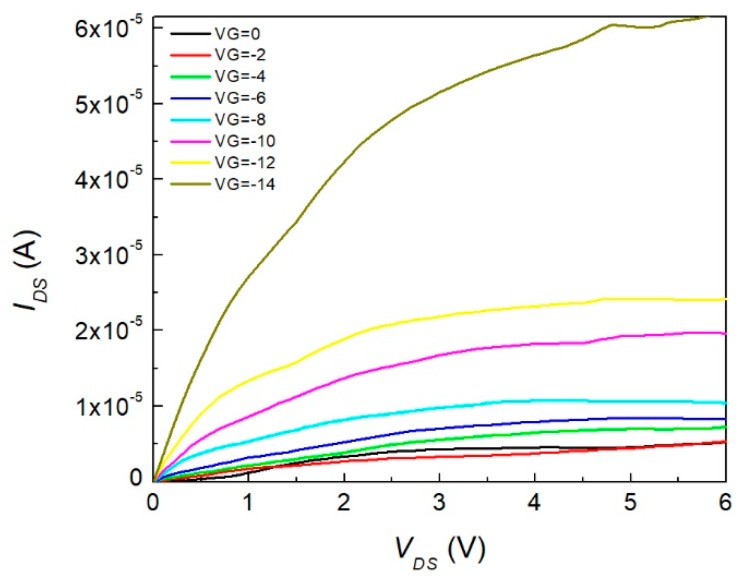
Voltage–current characteristics of the device.

We previously mentioned current–voltage characteristics were obtained for the device. The output characteristics of the InClPc OFET was conducted at room temperature using a gate-source polarization interval between 0 and −14 V, as shown in Figure 8, and a drain source voltage between 0 and 12 V. The drain current of the InClPc OFET increased when it was tested with negative gate voltages, which could be used for current amplification devices. By analysis of the curves obtained for the different tests, the OFET at small drain-source voltages (*V_DS_*) behaved within the linear region and at *V_DS_* values close to the gate voltages (*V_GS_*) in the saturation region. These characteristics indicated that the OFET had a p-type behavior, since by introducing a negative voltage in the electrode it produced such an increase in energy at HOMO and LUMO orbitals of the semiconductor InClPc. When the HOMO aligned with the Fermi levels of contacts, the electrons went from the InClPc to the contacts, leaving holes positively charged. The application of a negative potential caused these holes to move to the collector as charge carriers.

Charge mobility and threshold potential were the parameters used to characterize an OFET device. Such parameters were extracted from curves in Figure 8 by considering that the current was controlled by two independent and perpendicular voltages, and the voltage was applied to the gate electrode and between the source and collector electrodes. Charge mobility was calculated using reported methodology by Bittle et al. using the following equations, where Equation (1) was used for the linear mode and Equation (2) was used for the saturation mode in the device’s operation regime.

For the linear mode that meets the condition: $\left|V_{DS}\right|<\left|V_{GS}-V_{th}\right|$,

(1) $$I_{D}=\mu_{lin}C_{ox}\frac{W}{L}\left[\left(V_{GS}-V_{th}\right)V_{DS}-\frac{V_{DS}{}^{2}}{2}\right].$$

For the saturation mode that meets the condition: $\left|V_{DS}\right|>\left|V_{GS}-V_{th}\right|$,

(2) $$I_{D}=\mu_{sat}C_{ox}\frac{W}{2L}{\left(V_{GS}-V_{th}\right)}^{2},$$

where *V_th_* is the threshold voltage, *V_GS_* is the gate voltage, *V_DS_* is the drain voltage, *I_D_* is the drain current, $\mu_{lin}$ is the linear mobility, $\mu_{sat}$ is the saturation mobility, $C_{ox}$ is the oxide capacitance per unit area, and *L* and *W* are the length and width of the transistor channel [33]. Based on the behavior of the device, we noticed that the InClPc OFET worked in the linear mode charge mobility because the device met the condition $\left|V_{DS}\right|<\left|V_{GS}-V_{th}\right|$ for low *V_DS_*. In the linear region, the OFET operated as a voltage-controlled resistor. Besides the output characteristics of the OFET device, it was also important to analyze the device’s current parameters such as the mobility. In order to get the mobility for the fabricated device, an indium (III) phthalocyanine capacitance value of 3.5 × 10^15^ F^−2^, reported by Zeyada et al., was selected [34]. The drain and source evaporated contacts were 1 × 1 mm^2^ square shape with a separation of 3 mm between them. To estimate the mobility of the OFET, we used *C* = 5.67 × 10^−7^ F, *L* = 0.3 cm, and *W* = 0.1 cm. Field effect mobility was calculated from Equation (1), and the obtained maximum value was 36.2 cm^2^ V^−1^ s^−1^ (*V_DS_* = 1.2, *V_GS_* = −1.4), which was superior to the mobility values obtained in conventional OFETs manufactured with poly(3-hexiltiofeno) [35] and pentacene [36]. It should be taken into account that 0.1 cm^2^ V^−1^ s^−1^ mobilities are needed to compete with organic semiconductors, which brings attention to the obtained device [37,38]. In Figure 8, the square root of the current across the drain-source against *V_GS_* was observed for a constant *V_DS_* = 1.2 V. A linear region was observed for gate-source voltages greater than −5 V, and a starting square root current value of 1.33 A^1/2^ was observed when *V_GS_* was equal to zero and increased around three times as the voltage varied to −12 V. The latter was directly related to the on/off ratio of the OFET, which showed a prominent current change when applying small gate voltages. For the selected *V_DS_* there was a large on/off ratio. Nevertheless, augmenting *V_DS_* to higher values increased the device’s output response.

**Figure 9:**
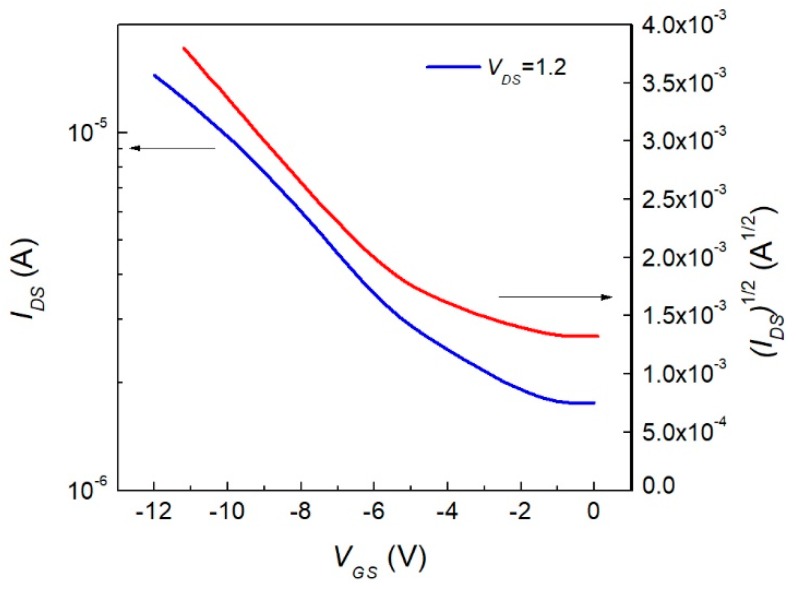
Flexible OFET output and current–voltage characteristics.

**Figure 10:**
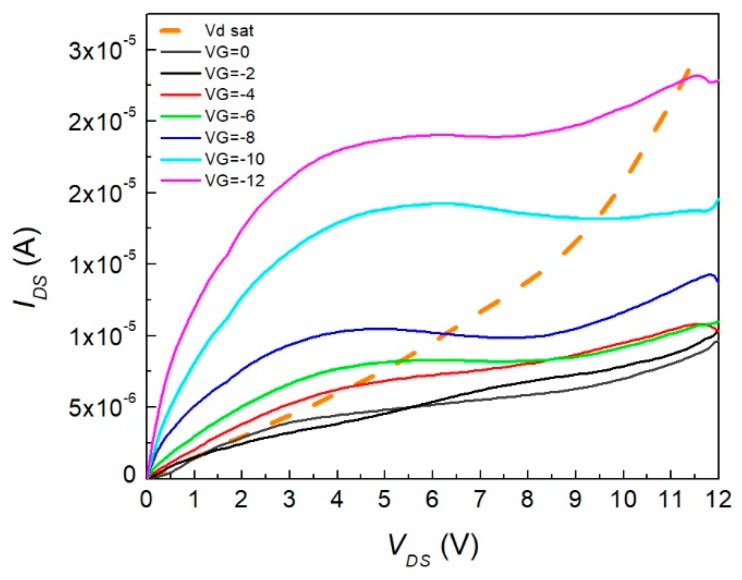
Threshold voltage characterization of the device.

In Figure 9, the OFET device current–voltage characteristics are depicted. The threshold voltage was obtained from the linear extrapolation intercept with the abscissa axis. The section had a threshold voltage value of 0.59 V. Therefore, *V_GS_* values greater than this allowed conduction between the source and drain. This threshold voltage was calculated with a voltage of 1.2 V applied on the source-drain. The value was positive, which was comparable to those of CuPcs [8]. Also, it whenever *V_GS_* increased, the characteristic region seemed to have a greater pronunciation with a larger charge current mobility. The OFET subthreshold swing (SS) was calculated from Figure 9 and determined to be a p-type semiconductor material, as stated previously, with a result of 80.64 mV/dec. This value was related to the key limiting factor of the FET by diffusion of charge carriers, which was greater than that of inorganic FETs at room temperature. It was desirable that this value decreased to lower values than 60 mV/dec; this could increase the current that the device could drive and, furthermore, could be adjusted with the channel dimensions. Figure 10 shows the saturation voltage (*V_sat_*) as a dashed curve mounted on *I_DS_*–*V_DS_* characteristics for the different applied gate voltages. The device operated when *V_DS_* was lower than that of *V_sat_*. Furthermore, when *V_G_* increased to negative values, *V_sat_* increased. So, for lower *V_G_* values, the device’s operating current and voltage values were limited. Nevertheless, this is desirable for commuter devices with on/off switches, as for low *V_DS_* it is possible to make the state transition.

**Figure 11:**
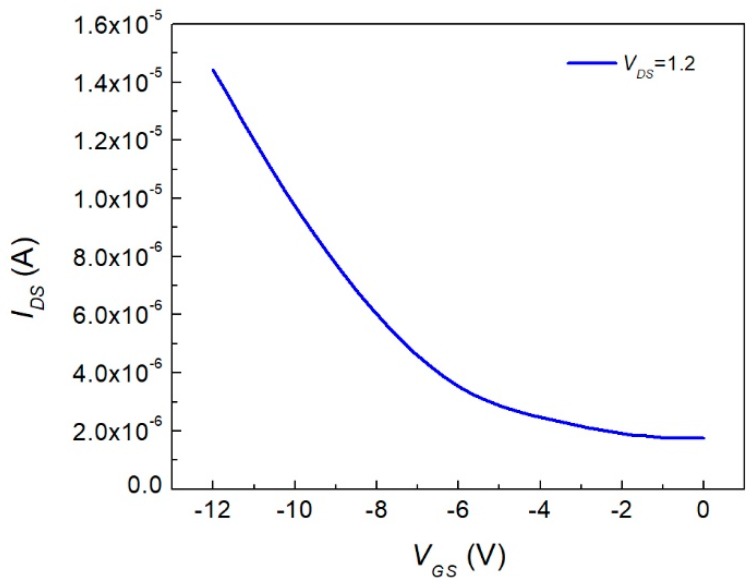
Square root current–voltage characteristics and slopes for the OFET.

In Figure 11, we can observe how the OFET´s current increased when a higher *V_GS_* was applied. This representation was done with a constant 1.2 V acting on the *V_DS_* and by varying the *V_GS_* from 0 to −14 V. It was important to observe that when *V_GS_* was equal to zero, the device presented a current on the order of microamperes, while when *V_GS_* was −14 V, the device presented a current on the order of dozens of microamperes, which increased by one order of magnitude. Also, as seen in the curve, the device started to work when *V_GS_* surpassed *V_th_*. At about 0.59 V, the device started to have an increasing current.

**Figure 12:**
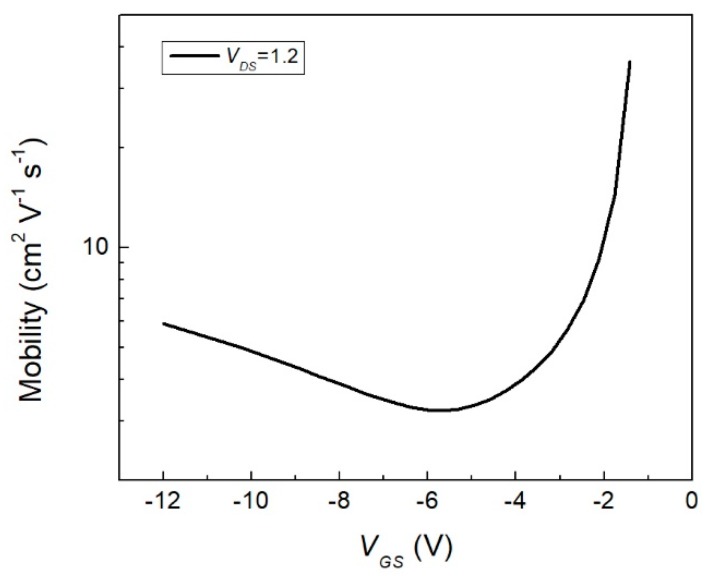
Linear mobility characteristics as function of the gate bias.

In Figure 12, the *V_GS_* dependent mobility of the OFET device is shown on a logarithmic scale. The device became functional when it surpassed its *V_th_* of 0.59 V. The maximum mobility of the device was obtained when *V_GS_* was closest to the *V_th_* region. At this point, the device had a mobility of 36.2 cm^2^ V^−1^ s^−1^. As the *V_GS_* applied to the device started increasing to higher values, the mobility had a pronounced decrease until minimum mobility was reached. The device had a minimum mobility of about 3.2 cm^2^ V^−1^ s^−1^ with a *V_GS_* of −5.6 V. The device offered a fast response in regard to its mobility, which is a primary characteristic pursued in high-response electronic devices. In an interval of ~3.2 V in *V_GS_* (from −1.4 to −5.6 V) the device changed the carrier mobility ~33 cm^2^ V^−1^ s^−1^ (from 36.2 to 3.2 cm^2^ V^−1^ s^−1^), which was an important change in mobility for a small *V_GS_* interval. This gave us a ratio mobility of 11.32 with a *V_GS_* interval of 3.2 V. However, when the *V_GS_* started to increase further, the device´s mobility started increasing again but at a lower rate. When *V_GS_* was −12 V, the device had a second maximum mobility, which had a value of 5.9 cm^2^ V^−1^ s^−1^. The improved mobility was a consequence of the bilayer structure and was boosted by the presence of the indium(III) phthalocyanine chloride. The latter was because the InClPc behaved as a p-type semiconductor that had high electron mobility in the p electronic orbital and in their delocalized bonds and π-type bonds. On the other hand, using a bilayer gate dielectric increased the mobility of OFETs devices. It was important to notice that this behavior was closely related with the amorphous structure, which caused significant improvements in FET mobility. Thermal annealing helped to improve device performance and film homogeneity. Nevertheless, results show that the fabricated flexible organic FET present adequate and improved device operative properties, and it can be used in several application fields where flexible materials are necessary.

## 4. Conclusions

A flexible OFET with a sandwich bottom-gate/top-contact structure configuration was manufactured using a high-vacuum evaporation technique. The film of the dielectric constituted Al_2_O_3_ particles embedded in a nylon 11 polymeric matrix to enhance the operation of the device. FTIR spectroscopy verified that the thin films that integrated the device did not suffer degradation during the deposition process. SEM images evidenced homogeneous deposition of nylon. On the other hand, there were no impurities that affected charge mobility in the OFET. A band gap of 1.67 eV was obtained using Tauc’s model for indirect transitions, corresponding to amorphous structures. This value was in the inferior limit of the band gap for organic semiconductors, which increased the potential of the OFET to be used in flexible organic electronics. From the evaluation of voltage–current characteristics in the device, a maximum charge mobility of 36.2 cm^2^ V^−1^ s^−1^ was achieved, which was superior to that reported by similar OFETs materials. We observed that the use of a bilayer structure enhanced OFET electrical performance, such as device mobility. Also the drain current of the InClPc-OFET increased when it was tested with negative gate voltages. By analyzing the curve obtained for the different tests, the OFET behaved within the linear region at small drain-source voltages. The flexible OFET presented adequate and improved device operative properties. It can be used in several application fields where flexible materials are necessary. As observed, it also offers a fast response regarding its mobility, which is desirable in high-response electronic devices. Because of its pronounced change in *V_sat_* and ability to change its current by applying small gate voltages, the device could be used in amplification and in on/off gateway applications.

## Figures and Tables

**Table 1:** Fluorescent density (%) for the different OFET micrographs.

OFET Region (Figure 4)	Fluorescent Density (%)
Region 1	2.8
Region 2	5.6
Region 3	0.6
Region 4	0.9

Organic field-effect transistor, OFET.
